# GEO dataset mining analysis reveals novel *Staphylococcus aureus* virulence gene regulatory networks and diagnostic targets in mice

**DOI:** 10.3389/fmolb.2024.1381334

**Published:** 2024-03-28

**Authors:** Guangyu Xu, Yue Yang, Yan Lin, Yu Bai

**Affiliations:** ^1^ College of Pharmacy, Beihua University, Jilin, China; ^2^ School of Basic Medical Sciences, Beihua University, Jilin, China; ^3^ College of Pharmacy, Jilin Medical University, Jilin, China

**Keywords:** *Staphylococcus aureus*, pathogenic gene, transcription factor, regulatory network, network node

## Abstract

*Staphylococcus (S.) aureus* infection is a serious, worldwide health concern, particularly in many communities and hospitals. Understanding the *S. aureus* pathogenetic regulatory network will provide significant insights into diagnostic target screening to improve clinical treatment of diseases caused by *S. aureus*. We screened differentially expressed genes between normal mice and *S. aureus*-infected mice. We used the Gene Expression Omnibus (GEO) DataSets database for functional analysis (GO-analysis) and the DAVID and KEGG databases for signaling pathway analyses. We next integrated the gene and pathway analyses with Transcriptional Regulatory Element Database (TRED) to build an antimicrobial resistance gene regulatory network of *S. aureus*. We performed association analysis of network genes and diseases using DAVID online annotation tools. We identified a total of 437 virulence genes and 15 transcription factors (TFs), as well as 444 corresponding target genes, in the *S. aureus* TF regulatory network. We screened seven key network nodes (*Met*, *Mmp13*, *Il12b*, *Il4*, *Tnf*, *Ptgs2*, and *Ctsl*), four key transcription factors (*Jun*, *C3*, *Spil*, and *Il6*) and an important signaling pathway (TNF). We hypothesized that the cytokine activity and growth factor activity of *S. aureus* are combinatorically cross-regulated by *Met*, *Mmp13*, *Il12b*, *Il4*, T*nf*, *Ptgs2*, and *Ctsl* genes, the TFs *Jun*, *C3*, *Spi1*, and *Il6*, as well as the immune response, cellular response to lipopolysaccharide, and inflammatory response. Our study provides information and reference values for the molecular understanding of the *S. aureus* pathogenetic gene regulatory network.

## 1 Introduction

Infection caused by *Staphylococcus (S.) aureus* is an endemic health problem worldwide (Chen et al., 2022; Park et al., 2021; Miller et al., 2020). *S. aureus* is a common invasive bacterial pathogen that produces staphylococcal enterotoxin (SE), which causes intestinal tract dysfunction (Dicks et al., 2021; Omar et al., 2021) and is responsible for almost all staphylococcal food poisoning. Staphylococcal food poisoning results from food contamination by *S. aureus* enterotoxin, accounting for 33% of total bacterial foodborne infections in the United States (Haghi et al., 2021). More than 45% of foodborne diseases are caused by *S. aureus* in Canada (Greco et al., 2020). Therefore, *S. aureus* pathogenicity and its underlying virulence mechanisms have been a primary research focus. *Staphylococcus aureus* causes high mortality, which is associated with early excessive inflammation of unknown mechanisms (Wang et al., 2023). *Staphylococcus aureus* has a powerful virulence secretion system to evade the host’s immune response, and may even promote excessive inflammatory response (Mu et al., 2023); therefore, the host’s regulation of the immune response, especially the key mechanisms controlling inflammation, is crucial for successful resistance to *Staphylococcus aureus*.

A pathogenic gene permits a pathogen to cause disease (Kotnik et al., 2023). Understanding the role of virulence genes in disease has become a central focus of medical research for the purpose of developing preventive measures, diagnostic tools, treatment approaches, and therapeutic strategies (Jonas et al., 2020). Previous studies on *S. aureus* pathogenicity have been mainly focused on the expression and function of a single gene using gene knockout, gene silencing, RNA interference, and other genetic approaches (Kane et al., 2018; Scherr et al., 2015). However, these methods are typically laborious and time-consuming, inefficient, and require extensive training, leading to limited success in meeting the needs of clinical medicine. Advanced omics technologies, including gene chip and big data analytics (Giulieri et al., 2020) particularly CRISPR/CAS9 (Chen et al., 2017; Penewit et al., 2018), can simultaneously identify nearly one million sites in genomic DNA, which allows for association analysis between *S. aureus-*infected diseases and genetic variation. Thus, these technologies provide powerful tools to investigate pathogenic gene regulatory networks and diagnostic targets. However, scientists are challenged by the increasing amounts of transcriptomic data created by high-throughput techniques, including how best to handle and analyze the millions of data points identified by genetic studies of *S. aureus-*infected diseases with appropriate mathematical and statistical strategies.

The objective of this study was to identify novel *S. aureus* pathogenic gene regulatory networks and diagnostic targets using the NCBI GEO DataSets database and functional enrichment analysis. In the present study, the gene expression profiles of *S. aureus*-infected mice were selected from the NCBI GEO Datasets database to assess differentially expressed genes (DEG) using a combination of the linear models and empirical Bayesian methods in *limma*, an R software package (https://www.r-project.org/), with the traditional t-test. Gene function analysis (GO-analysis) and signal pathway analysis (Pathway-Analysis) were performed using DAVID (Database for Annotation, Visualization and Integrated Discovery) and KEGG (Kyoto Encyclopedia of Genes and Genomes) to select DEG sets that were integrated into TRED (Transcriptional Regulatory Element Database) to construct the *S. aureus* antimicrobial resistance gene regulatory network. Associations between a given disease and network genes were analyzed using the DAVID online annotation tool.

## 2 Experimental methods

### 2.1 Chip data

Chip data were pooled from the NCBI GEO DataSets database (https://www.ncbi.nlm.nih.gov/gds) using the keywords (*Staphylococcus aureus*) AND “*Mus musculus*” [porgn: txid9606], while data only from *S aureus*-infected mice and gene chips with Affymetrix CEL files were adopted (Davis et al., 2007).

### 2.2 Chip data processing

First, we performed background correction on the chip data, and then the probe fluorescence values were converted into gene expression values using the Expression Console™ software tool (Affymetrix, Santa Clara, California, United States) (www.affymetrix.com). The chip data were logged and normalized by Transcriptome Analysis Console (Affymetrix, Santa Clara, California, United States). Differentially expressed mRNAs were compared between the normal and *S. aureus*-infected mice using SAM (Significance Analysis of Microarray) (http://www-stat.stanford.edu/∼tibs/SAM/index.html). DEGs with fold change > 2.0 or fold change < −2.0 and a *p*-value < 0.05 were selected for further study. DEGs overlapping in two or more platforms were further screened with Venn diagrams to account for differences among the chip platforms (Lu et al., 2007).

### 2.3 Transcription factor (TF) and corresponding target gene screening

We first selected the “Search TF Target Genes” option in http://rulai.cshl.edu/TRED. Next, we selected Factor Name in the Type of search key option and entered the Gene symbol name. In the third step, we selected *Mus musculus* in Target Gene Organism and selected “all” at Promoter Quality and Binding Quality. At the final step, we searched for corresponding target genes (Zhao et al., 2005).

### 2.4 Gene co-expression network

We identified a total of 15 TFs and their predicted corresponding target genes. A total of 444 target genes were paired to analyze TF-to-target regulatory relationships. Differential co-expression correlations between gene pairs were estimated by differential co-expression analysis (DCEA) (Markus et al., 2017) and then mapped to mouse TF-to-target pairs to identify TF-gene transcriptional regulatory pairs that were visualized using Cytoscape software (Sun et al., 2017).

### 2.5 GO function annotation analysis

A set of 437 genes were submitted to the DAVID database (http://david.abcc.ncifcrf.gov/) for enrichment analysis (Huang et al., 2007) of DEG sets with the Functional Annotation Tool, where OFFICIAL_GENE_SYMBOL was selected and the whole genome of *Mus musculus* was used as the background genes.

### 2.6 Significance analysis of DEGs

DEGs were annotated based on the NCBI GO database (http://www.geneontology.org/). The significance level and misjudgment rate of each GO were estimated by Fisher’s exact test and chi-squared test (χ^2^), and the *p*-value was calibrated with the misjudgment rate to determine significance (*p* < 0.05) of GOs. The significant GOs (*p* < 0.05) were manually selected using the European Bioinformatics Institute (EBI) database (https://www.ebi.ac.uk/) (Ashburner et al., 2000).

## 3 Experimental results

### 3.1 Chip data

In the present study, chip data were pooled from the NCBI GEO DataSets database using both *S aureus*-infected mice and Affymetrix gene chips with CEL files as the selection criteria. Three platforms of gene chips were selected and the detailed information is listed in Table 1.

**TABLE 1:** *S. aureus* GEO chip data information.

Dataset ID	Sample ID	Sample number	Platforms	Organism	Organ	Manufacturer
GSE25244	GSM621138-GSM621140	6	GPL1261	*Mus musculus*	kidney	Affymetrix
	GSM621144-GSM621146					
GSE28540	GSM706724-GSM706728	10	GPL6246	*Mus musculus*	kidney	Affymetrix
	GSM706734-GSM706738					
GSE60088	GSM1464839-GSM1464842	8	GPL1261	*Mus musculus*	kidney	Affymetrix
	GSM1464844-GSM1464847					

Three sets of chips containing 6, 10, and 8 chips, respectively. Each set of chips includes two groups: normal and infected.

### 3.2 Chip data processing

**FIGURE 1:**
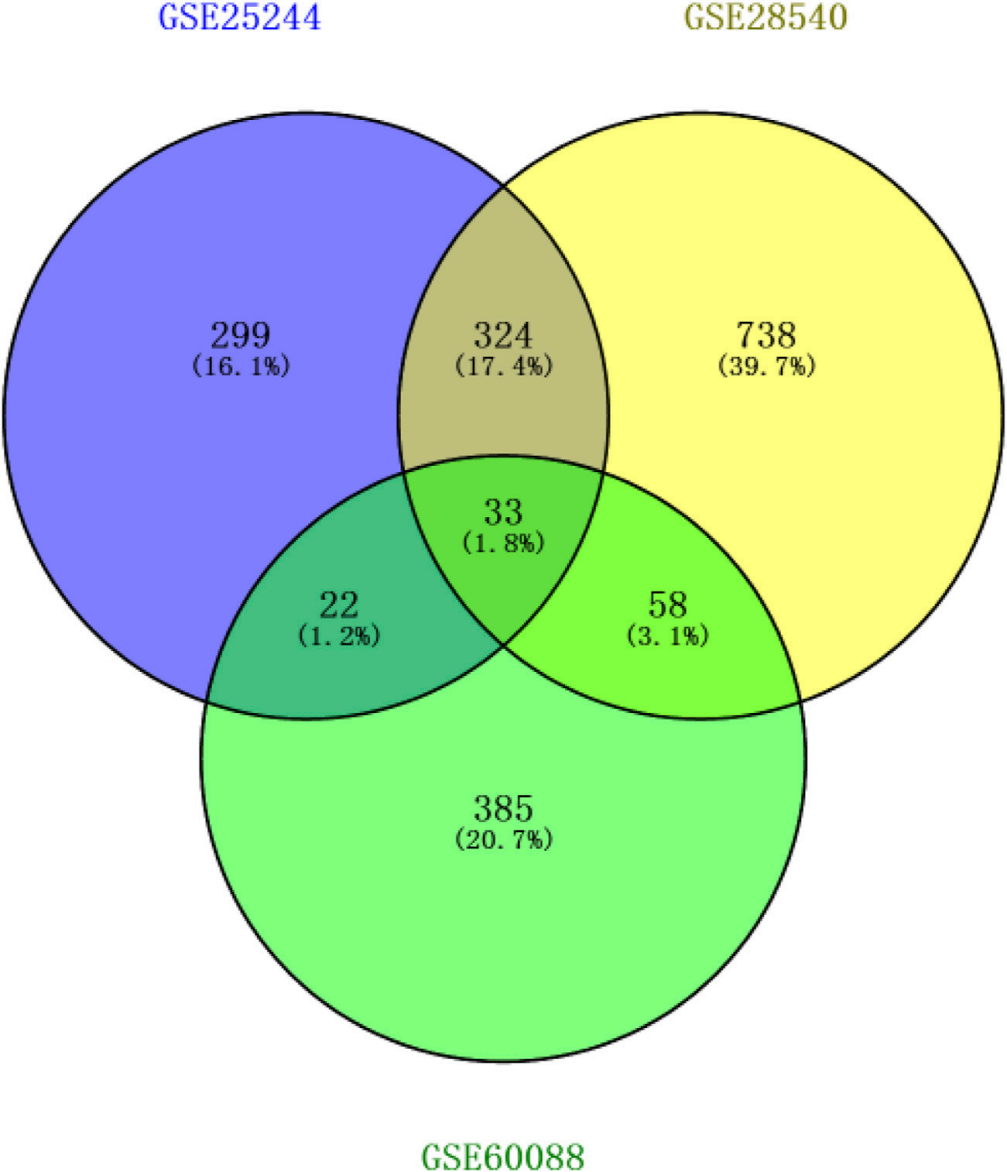
Screening results of genes overlapping in two or more platforms (blue circle represents GSE25244, yellow circle represents GSE28540, and green circle represents GSE60088).

After background correction of chip data, the probe fluorescence values were converted into gene expression values using the Expression Console™ software tool and then logged and normalized by Transcriptome Analysis Console. 299, 738, and 385 DEGs were respectively identified from the three platforms by SAM to compare differentially expressed miRNAs between the normal and *S. aureus*-infected mice. DEGs were then cross-screened; 33 DEGs overlapped in the three platforms, and 324, 22, and 58 DEGs co-existed in two platforms, respectively (Figure 1). Therefore, a total of 437 DEGs that overlapped in more than two platforms were used in subsequent analyses.

### 3.3 TF regulatory networks

**FIGURE 2:**
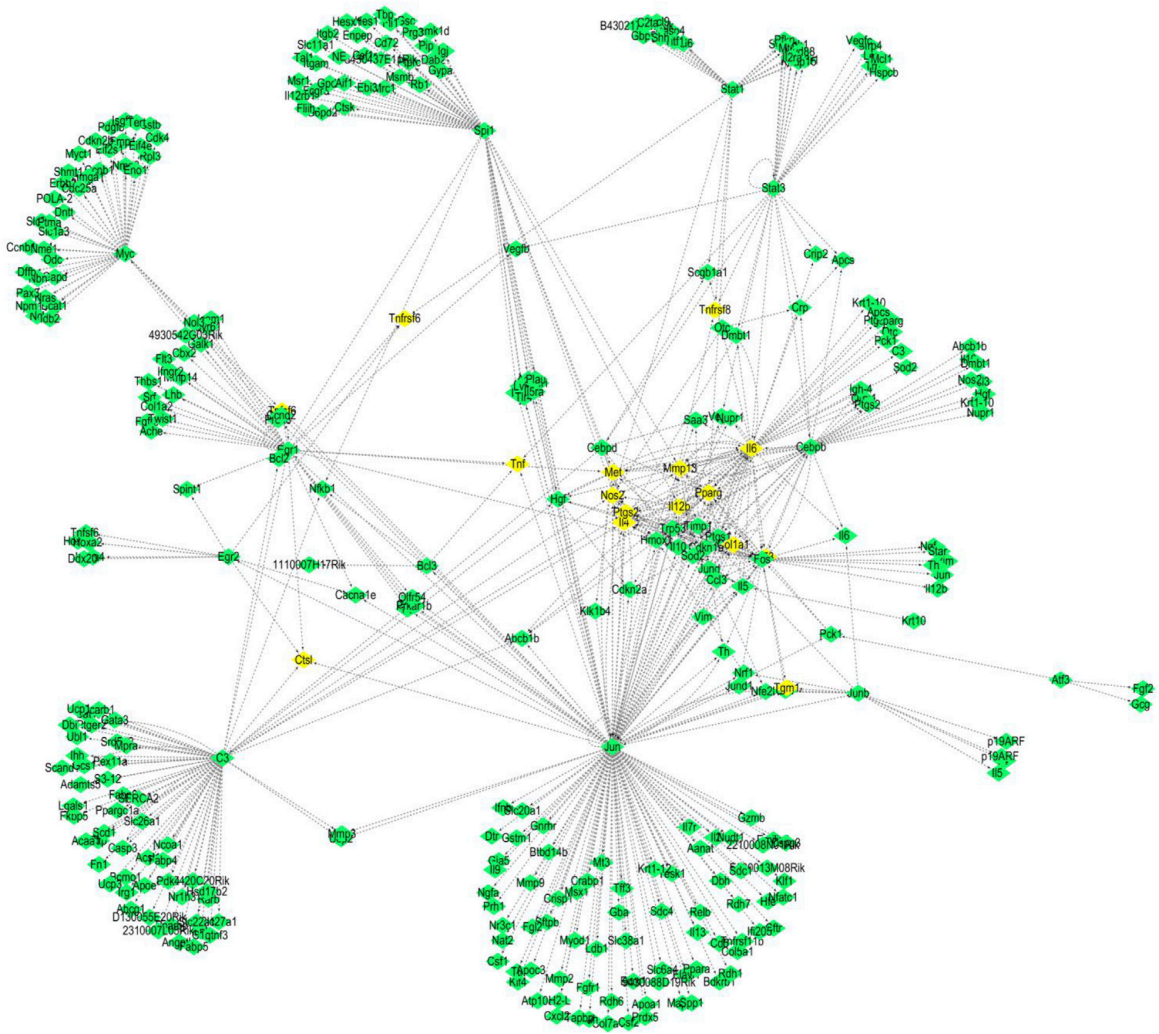
TF regulatory networks of 15 *S. aureus* virulence genes (yellow represents TFs; green represents corresponding target proteins).

We used the TRED database to predict possible TFs for 437 DEGs. We identified 15 TFs and 444 corresponding target genes (Table 2). As shown in Figure 2, we visualized these TFs and genes using Cytoscape to develop *S. aureus* pathogenic gene TF regulatory networks. This analysis demonstrated that 16 target genes were co-regulated by at least three TFs (Table 3). Among these, *me* was regulated by five TFs, and *mmp13*, *il12b*, *il4*, *tnf*, *ptgs2*, and *ctsl* were regulated by four TFs.

**TABLE 2:** 15 *S. aureus* pathogenic gene TFs and their corresponding target genes.

TF	Target gene NO.	Gene ID	Description
Jun	120	16476	jun proto-oncogene
C3	62	12266	complement component 3
Spi1	49	20375	spleen focus forming virus (SFFV) proviral integration oncogene
Myc	38	17869	Myelocytomatosis oncogene
Egr1	31	13653	early growth response 1
Il6	30	16193	interleukin 6 receptor, alpha
Stat3	26	20848	signal transducer and activator of transcription 3
Stat1	21	20846	signal transducer and activator of transcription 1
Cebpb	20	12608	CCAAT/enhancer binding protein (C/EBP), beta
Fos	15	14281	FBJ osteosarcoma oncogene
Junb	11	16477	jun B proto-oncogene
Egr2	9	13654	early growth response 2
Cebpd	6	12609	CCAAT/enhancer binding protein (C/EBP), delta
Atf3	3	11910	activating transcription factor 3
Bcl3	3	12051	B cell leukemia/lymphoma 3

**TABLE 3:** Target genes regulated by *S. aureus* pathogenic gene TFs.

Target genes regulated by TFs	TFs regulating target genes	Regulated TFs
Met	5	Cebpb, Egr1, Il6, Jun, Spi1
Mmp13	4	Cebpb, Il6, Jun, Spi1
Il12b	4	Il6, Jun, Junb, Spi1
Il4	4	C3, Fos, Jun, Spi1
Tnf	4	Bcl3, Egr1, Jun, Stat3
Ptgs2	4	C3, Cebpd, Jun, Spi1
Ctsl	4	C3, Egr1, Egr2, Jun
Tgm1	3	Fos, Jun, Junb
Col1a1	3	Cebpb, Il6, Jun
Il6	3	Fos, Cebpb, Junb
Tnfrsf8	3	Jun, Stat1, Stat3
Tnfrsf6	3	Egr1, Egr2, Stat1
Tnfsf6	3	Egr1, Jun, Myc
Pparg	3	Cebpb, Cebpd, Stat1
p53	3	Cebpb, Il6, Jun
Nos2	3	C3, Jun, Stat1

Target genes regulated by TFs; TFs regulating target genes; Regulated TFs.

### 3.4 Analysis of network nodes in TF regulatory networks

As shown in Figure 2, we statistically recorded the network nodes in TF regulatory networks, finding 14 genes with more than 10 nodes. Among these genes, six (*ptgs2*, *trp53*, *mmp13*, *hmox1*, *il4*, and *pparg*) contained more than 15 nodes (Table 4). *Met*, *ptgs2*, and *mmp13* were regulated by five, four, and four TFs, respectively, showing close association with *S. aureus* virulence genes.

**TABLE 4:** Nodes in the *S. aureus* pathogenic gene regulatory network.

Gene	Network node	TFs regulating target gene
Ptgs2	19	4
Trp53	18	
Mmp13	17	4
Hmox1	17	
Il4	16	4
Pparg	15	3
Il12b	14	4
Timp1	14	
Jund	14	
Met	13	5
Nos2	12	3
Cdkn1a	11	
Il10	11	
Ptgs1	11	

Gene; Network node; TFs regulating target gene.

### 3.5 Annotation analysis of GO functions of DEGs

437 DEGs were annotated for GO functions (http://www.geneontology.org/), from which the top 10 pathways with the highest *p* values were further analyzed (Table 5). Ten pathways were primarily associated with disease-related pathways, among which the *S. aureus* infection pathway ranked first.

**TABLE 5:** GO function annotations of *S. aureus* DEGs.

Pathway	Gene NO	*p*-value	Benjamin
*Staphylococcus aureus* infection	24	1.3E-21	2.7E-19
Chemokine signaling pathway	39	1.3E-19	1.4E-17
TNF signaling pathway	28	4.9E-17	3.5E-15
Leishmaniasis	22	3.2E-16	1.8E-14
Rheumatoid arthritis	23	7.8E-15	3.3E-13
Tuberculosis	30	3.3E-13	1.2E-11
Phagosome	27	6.0E-11	1.8E-9
Pertussis	18	1.5E-10	3.9E-9
Osteoclast differentiation	22	5.8E-10	1.4E-8
Cytokine-cytokine receptor interaction	30	1.4E-9	2.9E-8

## 4 Discussion

*Staphylococcus aureus* predominantly resides on the skin and in the nasopharynx of humans, and it is the most prevalent cause of nosocomial and community-acquired bloodstream infections, skin and soft tissue infections, and pneumonia in almost all geographic areas. Thus, *S. aureus* poses a serious threat to human health and global stability. Methicillin-resistant *S. aureus* is unresponsive to 60% of antibiotics (Mitevska et al., 2021; Paudel et al., 2021), which is a major underlying cause of several difficult-to-treat life-threatening infections. Therefore, there is an unmet need to understand *S. aureus* pathogenesis to develop effective prevention and treatment of infection. In the present study, we identified 437 DEGs, from which 15 TFs and their predicted corresponding target genes were used to develop a TF regulatory network. We found several key factors closely related to inflammation and the immune system that are regulated by *S. aureus* TF regulatory networks. Our findings provide new information and reference values for virulence genes in the transcriptional regulation of *S. aureus* infection.

We hypothesized that inflammatory and immune system diseases caused by *S. aureus* are likely regulated by three genes: *jun*, *c3*, and *spil*. *Staphylococcus aureus* is the most common pathogen that causes inflammatory and immune system diseases, including a variety of suppurative (pus-forming) infections (Carrel et al., 2017), pneumonia, pseudomembranous colitis, pericarditis, and even sepsis (Agarwal et al., 2018). *Jun*, *c3*, and *spi1* are believed to be involved in inflammation and immune responses. *Jun* has been reported to inhibit inflammatory factors and participate in immune system regulation, which is supported by Xie et al. (2014) who showed that downregulation of *jun* decreased expression of pro-inflammatory cytokines such as tumor necrosis factor-α (TNF-α), interferon β (IFN-β), and interleukin 6 (IL-6), but upregulated expression of anti-inflammatory cytokines, including IL-10. *Jun* is also closely associated with systemic lupus erythematosus (SLE), an autoimmune disease involving multiple organs and systems (Maria et al., 2017). Doníz et al. (2011) reported that *jun* expression was significantly elevated in peripheral blood mononuclear cells (PBMC) in SLE patients compared to normal controls. C3, the most important molecule encoded by gene *c3* in the complement system (Lu et al., 2018), is located at the intersection node of both classical and alternative complement activation pathways, as well as the mannose-binding lectin pathway, indicating that C3 plays an important regulatory role in the complement system, inflammation, and the immune system (Yuan et al., 2024). The complex and diverse C3 cleavage fragments and their binding proteins regulate the complement system via activating the complement cascade and through self-activation and cleavage, as well as by interacting with a variety of relevant factors that promote immune adhesion and pathogen phagocytosis (Fagan et al., 2017). *Spi1* affects the immune system by regulating mature B cells in the spleen (Batista et al., 2017). In the present study, we first reported that *jun* (corresponding to 120 target genes), *c3* (corresponding to 62 target genes), and *spi1* (corresponding to 49 target genes) regulated most of the target genes, strengthening our hypothesis that *jun*, *c3* and *spi1* regulate inflammation and the immune system.

We further revealed that *jun* and *spil* are core genes in the *S. aureus* regulatory network, while *c3* plus *il6* are secondary core genes. Regulation of core genes in inflammation and immune system diseases is executed mainly by regulating target genes *met*, *mmp13*, *il12b*, *il4*, *tnf*, *ptgs2*, and *ctsl*. In the present study, we found that all of these genes were regulated by at least four TFs (Figure 2). *Met*, *mmp13*, *il12b*, *il4*, and *ptgs2* were regulated by *jun* and *spil*. *Met*, *mmp13*, and *il12b* were simultaneously regulated by *il6*. *Ctsl*, *ptgs2*, and *il4* were regulated by *jun* and *c3*. In addition to *jun*, *c3*, and *spil*, *il6* was has been shown to be involved in inflammatory and immune system diseases, where *il6* acted not only as a TF but also as a target gene regulated by a TF. A previous study revealed a close association of *il6* with the immune system (Leal et al., 1999) by activating proinflammatory and other cytokines in B cells, hepatocytes, hybridoma cells, and plasma cells to improve the body’s resistance to *S. aureus* infection. However, it has also been reported that *il6* inhibits the adverse effects of macrophages to IFN-γ responsiveness (Saleh et al., 2021). *Met*, *mmp13*, and *ctsl* are all related to cancer (Mo et al., 2017; Yang et al., 2017; Zhang et al., 2016). Further analysis should focus on understanding the molecular mechanism of the core genes that interact with TFs and their corresponding target genes in inflammation and the immune system (Gao et al., 2024).

This study provides evidence that immune functions, including immune response, cellular response to lipopolysaccharide, and the inflammatory response, are regulated by network nodes that contain *ptgs2*, *mmp13*, *il12b*, and *met*, together with TF *jun*. In the present study, we identified 14 genes in 10 nodes, of which only four genes (*ptgs2*, *mmp13*, *il12b*, and *met*) were regulated by four TFs (Table 4). In addition to *met*, which was regulated by most TFs, *ptgs2*, *mmp13*, and *il12b* significantly regulated TFs and network nodes. All network nodes were compared to previous studies (Kondo et al., 2000; Yamaguchi et al., 2005; Gokulnath et al., 2017; Utsugi et al., 2010; Cui et al., 2013) and we found that most of the network nodes were related on a certain level and strongly correlated with TF *jun*. The main GO terms of these network nodes are cytokine activity and growth factor activity, functioning immune response, cellular response to lipopolysaccharide, and inflammatory response. In addition, the most significant 10 pathways were associated with immune-related diseases, including *S. aureus* infection (Dusane et al., 2018), leishmaniasis (Jaton et al., 2016; Cui et al., 2017), rheumatoid arthritis (Insa et al., 2017), and tuberculosis (Ashtekar et al., 2016); however, immune-related TFs and information pathways, including the chemokine signaling (Joanna et al., 2017) and TNF signaling (Nandi et al., 2017) pathways, were closely associated with other diseases. Therefore, in general, these diseases and pathways were associated with immune function.

## 5 Conclusion

We successfully identified 437 DEGs from the GEO database to develop a TF regulatory network of *S. aureus*. We analyzed the genes met, mmp13, il12b, il4, tnf, ptgs2 and ctsl and transcription factors Jun, C3, Spil and il6 pathways, and found that most of these genes were on the TNF signaling pathway. At last, we hypothesized that *met*, *mmp13*, *il12b*, *il4*, *tnf*, and *ptgs2* function together with TFs *jun*, *c3*, *spil*, and *il6* to regulate inflammation and the immune system. The present study thus provides information and reference values for understanding the regulatory mechanisms of TFs and their network of *S. aureus* virulence genes.

## Data Availability

The original contributions presented in the study are included in the article/supplementary material, further inquiries can be directed to the corresponding authors.
